# Enhanced Ability of Oligomeric Nanobodies Targeting MERS Coronavirus Receptor-Binding Domain

**DOI:** 10.3390/v11020166

**Published:** 2019-02-19

**Authors:** Lei He, Wanbo Tai, Jiangfan Li, Yuehong Chen, Yaning Gao, Junfeng Li, Shihui Sun, Yusen Zhou, Lanying Du, Guangyu Zhao

**Affiliations:** 1 State Key Laboratory of Pathogen and Biosecurity, Beijing Institute of Microbiology and Epidemiology, Beijing 100071, China; helei_happy@126.com (L.H.); anatee@163.com (J.L.); chenyuehong.happy@163.com (Y.C.); lijunfeng2113@126.com (J.L.); sunsh01@163.com (S.S.); 2 Lindsley F. Kimball Research Institute, New York Blood Center, New York, NY 10065, USA; wtai@nybc.org (W.T.); ygao@nybc.org (Y.G.); 3 Institute of Medical and Pharmaceutical Sciences, Zhengzhou University, Zhengzhou 450052, China

**Keywords:** Coronavirus, MERS-CoV, receptor-binding domain, therapeutic antibodies, nanobodies, cross-neutralization

## Abstract

Middle East respiratory syndrome (MERS) coronavirus (MERS-CoV), an infectious coronavirus first reported in 2012, has a mortality rate greater than 35%. Therapeutic antibodies are key tools for preventing and treating MERS-CoV infection, but to date no such agents have been approved for treatment of this virus. Nanobodies (Nbs) are camelid heavy chain variable domains with properties distinct from those of conventional antibodies and antibody fragments. We generated two oligomeric Nbs by linking two or three monomeric Nbs (Mono-Nbs) targeting the MERS-CoV receptor-binding domain (RBD), and compared their RBD-binding affinity, RBD–receptor binding inhibition, stability, and neutralizing and cross-neutralizing activity against MERS-CoV. Relative to Mono-Nb, dimeric Nb (Di-Nb) and trimeric Nb (Tri-Nb) had significantly greater ability to bind MERS-CoV RBD proteins with or without mutations in the RBD, thereby potently blocking RBD–MERS-CoV receptor binding. The engineered oligomeric Nbs were very stable under extreme conditions, including low or high pH, protease (pepsin), chaotropic denaturant (urea), and high temperature. Importantly, Di-Nb and Tri-Nb exerted significantly elevated broad-spectrum neutralizing activity against at least 19 human and camel MERS-CoV strains isolated in different countries and years. Overall, the engineered Nbs could be developed into effective therapeutic agents for prevention and treatment of MERS-CoV infection.

## 1. Introduction

Middle East respiratory syndrome (MERS) coronavirus (MERS-CoV), an emerging infectious coronavirus, was first reported in humans in Saudi Arabia in 2012 [1]. Bats are a likely natural reservoir of this virus, and dromedary camels are an important intermediate [2,3,4,5,6]. Camels are an important mode of transportation, particularly in the Middle East, and this application of these animals contributes significantly to camel-to-camel and camel-to-human transmission of MERS-CoV [7,8]. In addition, MERS-CoV may also be transmitted between humans in community or hospital settings [9,10,11,12,13]. Since its first emergence, MERS-CoV has continued to infect humans with a high mortality rate (>35%) (http://www.who.int/emergencies/mers-cov/en/). This situation calls for a consistent effort to develop effective countermeasures, including therapeutic antibodies and vaccines, to prevent and treat MERS-CoV infection.

MERS-CoV spike (S) protein, an enveloped glycoprotein, plays a key role in viral infection, viral attachment, and viral entry [14,15]. It is composed of S1 and S2 subunits: the receptor-binding domain (RBD) in the S1 subunit mediates MERS-CoV binding to its cellular receptor, dipeptidyl peptidase 4 (DPP4), and the S2 subunit subsequently mediates viral and cell membrane fusion, leading to viral entry into target cells [16,17,18,19,20]. The RBD of MERS-CoV S protein contains a critical neutralizing domain fragment capable of inducing strong neutralizing antibodies, and it is therefore considered to be an important therapeutic and vaccine target [21,22,23,24,25,26].

Several monoclonal antibodies (mAbs) have been developed to prevent and treat MERS-CoV infection, and most of these agents are based on the RBD [26,27,28,29,30,31]. However, conventional IgG mAbs and antibody fragments often have complex structures and unstable behavior [32,33,34]. Consequently, anti-MERS-CoV therapeutic antibodies with strong stability and simplified structures would be clinically valuable.

Camelid heavy chain variable domains (VHHs), also termed nanobodies (Nbs), are derived from the variable domains of the camelid heavy chain-only antibodies (HcAbs). These antibodies have distinctive properties, including high binding affinity, strong specificity for target antigens, good tissue penetration, and intrinsic stability under harsh conditions, such as extreme pH values, proteases, chemicals, and high temperature. Accordingly, they represent promising therapeutic tools for the treatment of human diseases [35,36,37]. Moreover, because Nbs do not require paired light and heavy chain domains to maintain antigen-binding activity, they can be easily modified by protein engineering techniques without loss of functionality [38]. Several monomeric Nbs can be readily fused to form multivalent or multispecific constructs, thereby improving their binding affinity and functionality. For example, monomeric proteins have been engineered into dimeric or trimeric proteins [38,39]. Also, multidomain Nbs have been generated by linking different Nbs targeting influenza virus hemagglutinin protein; the resultant agents have greater breadth, avidity, potency, and cross-neutralizing activity against divergent influenza viruses than the parent molecules [40], demonstrating the feasibility of engineering Nbs targeting multiple epitopes to increase their activity. It is therefore worthwhile to attempt to generate multidomain Nbs with improved activity against other emerging and re-emerging infectious viruses.

Previously, by immunizing llamas with a recombinant MERS-CoV RBD protein, we generated a monomeric Nb (Mono-Nb, NbMS10) that targets the RBD of MERS-CoV S protein [41]. In this study, we constructed two oligomeric Nbs, including dimeric Nb (Di-Nb) and trimeric Nb (Tri-Nb), and compared them to the Mono-Nb in terms of their ability to bind RBD proteins, inhibit RBD–DPP4 receptor binding, and cross-neutralize MERS-CoV infection. In addition, to demonstrate the advantages of the oligomeric Nbs relative to conventional antibodies, we evaluated the stability of these Nbs under the extreme conditions mentioned above. Overall, our data show that the engineered oligomeric Nbs have been significantly improved from the standpoint of binding affinity to the RBD, inhibition of the RBD-DPP4 binding, and cross-neutralizing activity against divergent strains of MERS-CoV. They also maintained greater pH, protease, chemical, and thermal stability than their mAb counterparts.

## 2. Materials and Methods

### 2.1. Construction and Expression of MERS-CoV RBD-Specific Dimeric and Trimeric Nbs

Dimeric and trimeric Nbs specific for MERS-CoV RBD were constructed by linking two or three monomeric Nb (Mono-Nb: NbMS10) [41] with a GGGGS linker and a C-terminal His_6_ tag followed by insertion into the *Pichia pastoris* yeast secretory expression vector pPICZαA (Invitrogen, Carlsbad, CA, USA). The recombinant Nbs were expressed in *Pichia pastoris* GS115 cells and purified using Ni-NTA columns (GE Healthcare, Cincinnati, OH, USA).

### 2.2. SDS-PAGE and Western Blot

MERS-CoV RBD-specific Nbs were detected by SDS-PAGE and Western blot, as previously described [42,43]. Briefly, Nbs (3 μg) were resolved on 10% Tris-Glycine SDS-PAGE gels, followed by staining with Coomassie Brilliant Blue or transferring to nitrocellulose membranes. The membranes were further blocked overnight at 4 °C with PBST containing 5% non-fat milk, and incubated for 1 h at room temperature with goat anti-llama IgG antibody (1:3000, Abcam, Cambridge, MA, USA) and horseradish peroxidase (HRP)-conjugated anti-goat IgG antibody (1:1000, R&D Systems, Minneapolis, MN, USA). The treated membranes were further incubated with ECL Western blot substrate reagents (Abcam) and visualized using Amersham Hyperfilm (GE Healthcare). A MERS-CoV RBD-specific mouse mAb (MERS mAb) and a SARS-CoV RBD-specific mouse mAb (SARS mAb) [44] were included as controls.

### 2.3. ELISA

Binding between Nbs and MERS-CoV RBD proteins was detected by ELISA as previously described [42,45]. Briefly, ELISA plates were coated overnight at 4 °C with recombinant wild-type or mutant MERS-CoV RBDs containing a C-terminal human Fc tag. The plates were blocked with 2% PBST at 37 °C for 2 h, and sequentially incubated at 37 °C with serially diluted Nbs, goat anti-llama antibody (1:5000, Abcam), and HRP-conjugated anti-goat IgG antibody (1:3000, Abcam) for 1 h each. After washing, the plates were further incubated with substrate (3,3’,5,5’-tetramethylbenzidine, Sigma, St. Louis, MO, USA), and the reactions were stopped with 1 N H_2_SO_4_. Absorbance at 450 nm (A450) was measured by ELISA microplate reader (Tecan, Morrisville, NC, USA). To compare binding activity, the median effective concentration (EC_50_) was calculated as previously described [46].

### 2.4. Surface Plasmon Resonance (SPR)

Binding between Nbs and MERS-CoV RBD protein was detected using a BiacoreS200 instrument (GE Healthcare) as previously described [41]. Briefly, recombinant Fc-fused MERS-CoV RBD protein (5 μg/mL) was captured on a Sensor Chip Protein A (GE Healthcare), and recombinant His_6_-tagged NbMS10 Nb at various concentrations was flowed over the chip surface in 10 mM HEPES (pH 7.4), 150 mM NaCl, 3 mM EDTA, and 0.05% surfactant P20 buffer. The sensorgram was analyzed using the Biacore S200 software (GE Healthcare). A 1:1 binding model was fitted to the data.

### 2.5. Flow Cytometry

Inhibition of binding between MERS-CoV RBD and cell-surface hDPP4 receptor by Nbs was analyzed by flow cytometry as previously described [24]. Briefly, hDPP4-expressing Huh-7 cells were incubated at room temperature for 30 min with MERS-CoV RBD-Fc protein (20 μg/mL), with or without serially diluted Nbs. The cells were incubated for 30 min with FITC-labeled anti-human IgG antibody (1:50, Sigma), and then analyzed by flow cytometry. Percentage inhibition was calculated based on the fluorescence intensity of RBD–Huh-7 binding in the presence vs. absence of Nbs.

### 2.6. MERS-CoV Micro-Neutralization Assay

The neutralizing activity of MERS-CoV RBD-specific Nbs was initially measured by a live MERS-CoV-based neutralization assay, as previously described [28,45]. Briefly, MERS-CoV (EMC2012 strain, 100 TCID_50:_ median tissue culture infective dose) was incubated with Nbs at 37 °C for 1 h. The Nb/virus mixture was added to Vero E6 cells, which were then cultured for 72 h at 37 °C. The cytopathic effect (CPE) was observed daily. The neutralizing activity of the Nbs was reported as 50% neutralization dose (ND_50_). The Reed–Muench method was used to calculate the values of ND_50_ for each Nb [47].

### 2.7. MERS Pseudovirus Neutralization Assay

The cross-neutralizing activity of MERS-CoV RBD-specific Nbs was measured by pseudotyped MERS-CoV neutralization assay as previously described [24,45]. Briefly, 293T cells were cotransfected with a plasmid encoding Env-defective, luciferase-expressing HIV-1 genome (pNL4-3.luc.RE) and a plasmid encoding the MERS-CoV S protein. Pseudotyped MERS-CoV was harvested from culture supernatants 72 h after transfection, incubated with serially diluted Nbs at 37 °C for 1 h, and added to Huh-7 cells. After 72 h, the cells were lysed in cell lysis buffer (Promega, Madison, WI, USA), incubated with luciferase substrate (Promega), and assayed for relative luciferase activity by Tecan Infinite 200 PRO Luminator (Tecan). The ND_50_ of Nbs was calculated as previously described [46].

### 2.8. Detection of Nb Stability

The stability of Nbs with respect to changes in pH was evaluated by incubation in PBS at various pH values (5.0, 7.0, or 8.0) for 24 h at room temperature [48]. The stability of Nbs in the presence of chaotropic denaturants was evaluated by incubation in PBS containing a gradient of concentrations of urea (Sigma) for 24 h at 25 °C [49]. The stability of Nbs with respect to proteolysis was evaluated by incubation in 10 mM HCl buffer (pH 2.0) containing various concentrations of pepsin (Sigma) for 1 h at 37 °C [49,50]. The thermal stability of Nbs was evaluated by incubation in PBS at various temperatures (4 °C, 37 °C, and 60 °C) for 24 h [49]. Treated and non-treated Nbs were subjected to the MERS pseudovirus neutralization assay. MERS mAb and SARS mAb were included as controls.

### 2.9. Statistical Analysis

Statistical analysis was performed by Student’s two-tailed *t*-test using the GraphPad Prism statistical software (San Diego, CA, USA). *p* values lower than 0.05 were considered statistically significant. *, **, and *** indicate *p* < 0.05, *p* < 0.01, and *p* < 0.001, respectively.

## 3. Results

### 3.1. Construction and Characterization of MERS-CoV RBD-Targeting Dimeric and Trimeric Nbs

**Figure 1:**
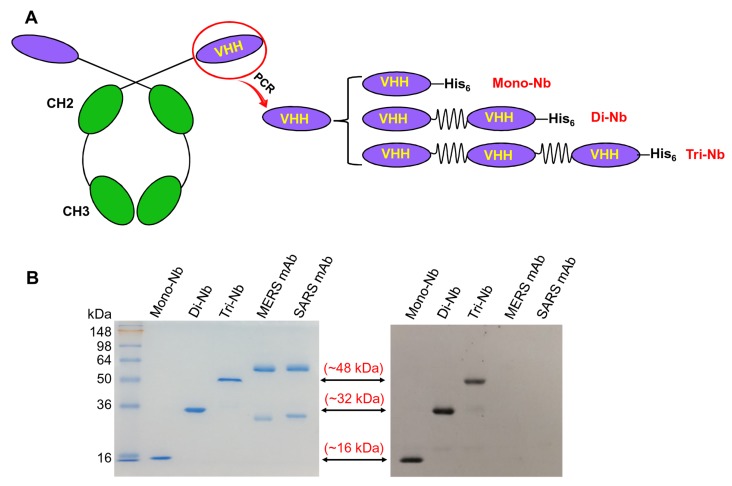
Construction and characterization of dimeric and trimeric nanobodies (Nbs) targeting the Middle East respiratory syndrome (MERS) coronavirus (MERS-CoV). (**A**) Heavy chain-only antibody (HcAb) consists of two constant heavy domains (CH2 and CH3) and heavy chain variable domains (VHHs). Monomeric Nb (Mono-Nb) was constructed previously by linking a MERS-CoV receptor-binding domain (RBD)-specific VHH and a C-terminal His_6_, and dimeric Nb (Di-Nb) and trimeric Nb (Tri-Nb) were constructed by linking two or three Mono-Nbs with GGGGS linkers and a C-terminal His_6_ tag for easy purification. (**B**) SDS-PAGE (left) and Western blot (right) analysis of MERS-CoV RBD-specific Nbs. The molecular weight marker (in kDa) is shown on the left. MERS-CoV RBD-targeting Mono-Nb was included as comparison, and MERS-CoV RBD-specific mAb (MERS mAb) and SARS-CoV RBD-specific mAb (SARS mAb) were used as controls. Anti-llama antibody was used for Western blot analysis.

HcAbs, presented in camelids and sharks, contain heavy chains but no light chains. The antigen-binding fragments of camelid HcAbs are also called VHHs (Figure 1A, left). Previously, using PCR to amplify the MERS-CoV RBD-specific VHH gene, we constructed a Mono-Nb targeting the MERS-CoV RBD. We linked this construct to a C-terminal His_6_ tag for easy purification [41], resulting in a total molecular weight of about 16 kDa. We generated the Di-Nb and Tri-Nb specific for MERS-CoV RBD by linearly linking two and three Mono-Nbs, respectively, with a flexible GGGGS linker between each Mono-Nb and a C-terminal His_6_ tag (Figure 1A, right). As with Mono-Nb, Di-Nb and Tri-Nb were expressed in culture supernatants of yeast expression cells at high yield and purity, and formed dimers and trimers with molecular weights of about 32 and 48 kDa, respectively (Figure 1B, left). These MERS-CoV RBD-targeting Nbs reacted strongly with an anti-llama antibody (Figure 1B, right). These data suggest that, like Mono-Nb, MERS-CoV RBD-specific Di-Nb and Tri-Nb maintained their native conformations and strong antigenicity.

### 3.2. MERS-CoV RBD-Targeting Dimeric and Trimeric Nbs Exhibited Superior Binding toward MERS-CoV RBD, Neutralization of MERS-CoV Infection, and Inhibition of RBD–hDPP4 Binding

**Figure 2:**
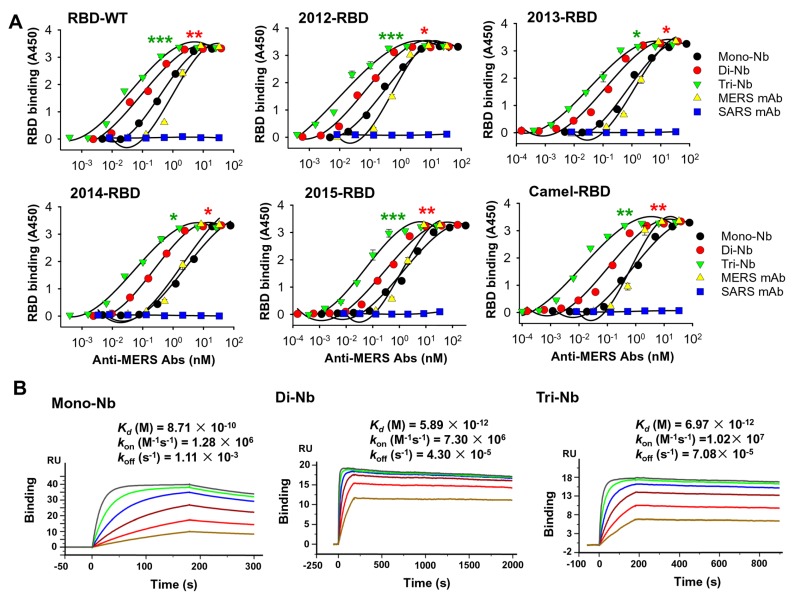
Detection of binding between MERS-CoV RBD-specific Nbs and MERS-CoV RBD proteins. (**A**) ELISA for binding between Di-Nb or Tri-Nb and RBD wild-type (RBD-WT) protein of the EMC2012 strain and mutant proteins containing RBD mutations from strains isolated from human and camel in 2012, 2013, 2014, and 2015. MERS-CoV RBD-targeting Mono-Nb was used for comparison, and MERS-CoV RBD-specific mAb (MERS mAb) and SARS-CoV RBD-specific mAb (SARS mAb) were included as controls. Data are presented as mean A450 ± standard error (s.e.m.) (*n* = 2). Experiments were repeated twice, yielding similar results. Significant differences in median effective concentration (EC_50_) ± s.e.m. were observed between Di-Nb and Mono-Nb, as well as between Tri-Nb and Mono-Nb, indicated by red and green asterisk (*, **, and ***), respectively. Concentration (in nM) was calculated based on predicted molecular weights of 16, 32, 48, and 150 kDa for Mono-Nb, Di-Nb, Tri-Nb, and mAb, respectively. (**B**) Surface Plasmon Resonance (SPR) analysis of binding between Di-Nb or Tri-Nb and RBD protein (i.e., RBD-WT). MERS-CoV RBD-targeting Mono-Nb was used for comparison. Binding parameters are shown in each figure.

To determine whether the engineered Nbs had stronger binding affinity to MERS-CoV RBD proteins, we performed ELISA to test their binding to wild-type MERS-CoV RBD protein fused to C-terminal hIgG1-Fc (RBD-WT), as well as Fc-fused RBD proteins containing mutations from MERS-CoV strains isolated from human and camel in 2012, 2013, 2014, and 2015 [24]. The results revealed that, relative to Mono-Nb, Di-Nb and especially Tri-Nb bound significantly more strongly to all RBDs tested; as expected, binding was dose-dependent (Figure 2A). In addition, the binding affinity of the MERS mAb control was similar to that of Mono-Nb, whereas the binding of the SARS mAb control was indistinguishable from background (Figure 2A). We then performed a SPR assay to test the binding affinity of these Nbs for RBD-WT. The results revealed that Mono-Nb, Di-Nb, and Tri-Nb had antibody binding affinity (*K_d_*) values of 0.87 nM, 5.9 pM, and 7 pM, respectively, toward RBD-WT (Figure 2B).

**Figure 3:**
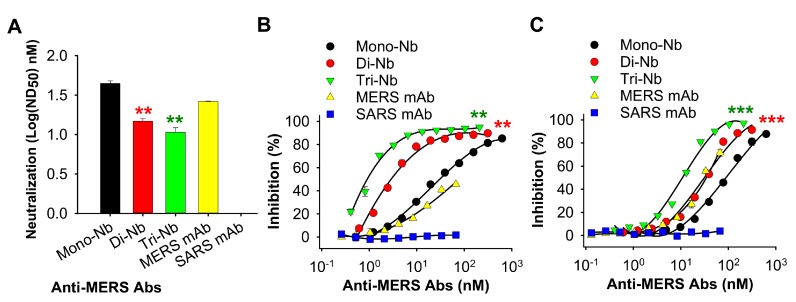
Detection of the neutralizing activity of MERS-CoV RBD-specific Nbs and their inhibition of RBD–DPP4 binding. (**A**) Neutralizing activity of Di-Nb and Tri-Nb against the prototypic MERS-CoV (EMC2012 strain). The neutralizing activity of the Nbs is expressed as the Nb concentration (nM) that completely inhibited the cytopathic effect (CPE) of MERS-CoV in at least 50% of the wells (50% neutralization dose: ND_50_). Data are expressed as mean ND_50_ ± s.e.m. (*n* = 3). (**B**) Inhibition of binding between Di-Nb or Tri-Nb and hDPP4 protein, as determined by ELISA. Percentage inhibition is expressed as RBD–hDPP4 binding in the presence or absence of Nbs based on the formula (1 – [RBD–hDPP4-Nb]/[RBD–hDPP4]) × 100. Data are presented as mean percentage inhibition ± s.e.m. (*n* = 2). (**C**) Inhibition of binding between Di-Nb or Tri-Nb and hDPP4-expressing Huh-7 cells, as determined by flow cytometry analysis. Percentage inhibition is expressed as RBD–Huh-7 binding in the presence or absence of Nbs, which is calculated based on the formula (1–[RBD–Huh-7–Nb]/[RBD–Huh-7]) × 100. Data are presented as mean percentage inhibition ± s.e.m. (*n* = 2). For (**A**)–(**C**), MERS-CoV RBD-targeting Mono-Nb was used for comparison, and MERS mAb and SARS mAb were included as controls. Experiments were repeated twice, yielding similar results. Significant differences among groups were compared by ND_50_ ± s.e.m. (**A**) or median inhibitory concentration (IC_50_) ± s.e.m. (**A**,**C**). Significant differences between Di-Nb and Mono-Nb are shown as red asterisk, and those between Tri-Nb and Mono-Nb are shown as green asterisk (** and ***).

We then performed a micro-neutralization assay to investigate the neutralizing activity of the engineered MERS-CoV RBD-specific Nbs against live MERS-CoV (EMC2012 strain) infection. The results revealed that both Di-Nb and Tri-Nb potently neutralized MERS-CoV infection with a significantly lower ND_50_ than Mono-Nb (Figure 3A). Previously, we demonstrated that the molecular mechanism of MERS-CoV RBD-specific Mono-Nb suppression of MERS-CoV involves inhibition of RBD–DPP4 receptor binding [41]. Here, we performed ELISA and flow cytometry assays to investigate whether the engineered Di-Nb and Tri-Nb could inhibit RBD-DPP4 binding to a greater extent than Mono-Nb. The ELISA result revealed that Di-Nb and Tri-Nb blocked the binding of MERS-CoV RBD (i.e., RBD-WT) to hDPP4 more strongly than Mono-Nb; the inhibition was dose-dependent (Figure 3B). The flow cytometry assay revealed that both Di-Nb and Tri-Nb had significantly greater ability than Mono-Nb to block the binding of RBD (i.e., RBD-WT) to Huh-7 cell-associated hDPP4; again, the inhibition was dose-dependent (Figure 3C). In both cases, the MERS mAb control inhibited RBD–DPP4 binding as well as or better than Mono-Nb, whereas inhibition by the SARS mAb control was indistinguishable from background (Figure 3B,C).

Taken together, the data described above suggest that, relative to monomeric Nb, MERS-CoV RBD-specific dimeric and trimer Nbs exhibited significantly improved binding affinity toward MERS-CoV RBD proteins, elevated neutralizing activity toward MERS-CoV infection, and more potent inhibition of MERS-CoV RBD binding to the DPP4 receptor.

### 3.3. MERS-CoV RBD-Targeting Nbs Maintain Strong pH, Protease, Chemical, and Thermal Stability

Nbs generally have intrinsic stability under a variety of extreme conditions, including low or high pH and temperatures, exposure to proteases (such as pepsin), and chaotropic agents (such as urea) [36,37]. To investigate the stability of MERS-CoV RBD-targeting Nbs, we subjected them to these extreme conditions, and then tested their neutralizing activity against pseudotyped MERS-CoV expressing the S protein of strain EMC2012. As shown in Table 1, all Nbs, including Mono-Nb, Di-Nb, and Tri-Nb, were still able to neutralize pseudotyped MERS-CoV infection after treatment at three different pH values (pH 5.0, 7.0, and 8.0), various concentrations of pepsin and urea, and three temperatures (4 °C, 37 °C, and 60 °C). All samples maintained neutralizing activity similar to their respective untreated counterparts. Although MERS mAb control, treated or not treated at the three pH values, maintained similar neutralizing activity against pseudotyped MERS-CoV infection, it significantly lost neutralizing ability after incubation with urea or pepsin, or after pre-treatment at 37 °C or 60 °C. As expected, the SARS mAb control had no cross-neutralizing activity against MERS-CoV after any of these treatments. These data indicate that, relative to traditional mAbs targeting MERS-CoV RBD, MERS-CoV RBD-specific Nbs maintain greater stability under all extreme conditions tested.

### 3.4. MERS-CoV RBD-Targeting Dimeric and Trimeric Nbs Had Significantly Elevated Cross-Neutralizing Activity Against Multiple Heterologous MERS-CoV Isolates

MERS-CoV has undergone a number of mutations, including those in the RBD [24]. Hence, it is critical that MERS-CoV RBD-specific Nbs maintain potent cross-neutralizing activity against MERS-CoV of divergent strains. In addition to pseudotyped MERS-CoV expressing the S protein of the prototypic MERS-CoV strain (EMC2012), we constructed 18 additional pseudotyped MERS-CoVs containing RBD mutations from MERS-CoVs isolated from seven countries (Saudi Arabia, UK, Qatar, Oman, Jordan, South Korea, and UAE), different time periods (2012–2016), and two hosts (human and camel) (Table 2). In addition, we tested the neutralizing activity of MERS-CoV RBD-specific Nbs. As shown in Table 2, relative to Mono-Nb, Di-Nb, and especially Tri-Nb, had significantly elevated neutralizing activity against all these 18 viruses tested. ND_50_ ranged from 0.81 to 27.1 nM for Mono-Nb, from 0.07 to 3.29 nM for Di-Nb, and from 0.01 to 0.61 nM for Tri-Nb. The neutralizing activity of MERS mAb was much lower than that of Di-Nb and Tri-Nb against all MERS-CoV strains tested, and SARS mAb had no neutralizing activity against these viruses. These data indicate that MERS-CoV RBD-specific oligomeric Nbs exhibited higher levels of cross-neutralization activity against divergent MERS-CoV strains.

## 4. Discussion

MERS-CoV continues to pose a severe threat to public health worldwide due to its high mortality rate and the steady increase in clinical cases, particularly in Saudi Arabia. Currently, no MERS vaccines or therapeutics have been approved for use in humans, creating an urgent demand for efficacious vaccines and therapeutic agents capable of preventing MERS-CoV transmission and infection, as well as treating MERS-CoV-infected humans and camels. In terms of antibody therapy, antibodies with high productivity, good antigen-binding affinity, and potent neutralizing activity against divergent strains of MERS-CoV infection would be of the greatest practical use.

Unlike conventional IgG antibodies (~150 kDa), or antibody fragments such as antigen-binding fragment (Fab, ~55 kDa) and single-chain variable fragment (scFv, 28 kDa), Nb monomers generally exhibit excellent solubility, strong stability, and good tissue penetration, mainly due to their small size (~15–16 kDa) [32,33,51]. In some cases, to increase efficacy against viral infection, bispecific, multispecific, or multivalent Nbs can be constructed by tandemly linking two or more Nb monomers recognizing either the same or different epitopes [52]. In contrast to mAb fragments, which might exhibit reduced expression, stability, or affinity after engineering or recombination, engineered Nbs exhibit elevated antigen-binding affinity, thus extending the time period during which Nbs are bound to their targets, while maintaining their beneficial characteristics, without negatively affecting production yields, solubility, or stability [53,54,55].

Taking advantage of the properties of Nbs, especially their ability to form functional bi- or multi-specific Nbs with elevated activity, we constructed two MERS-CoV RBD-targeting oligomeric Nbs, Di-Nb and Tri-Nb, based on the previously developed Mono-Nb [41], with the goal of identifying anti-MERS-CoV Nbs with improved binding ability, superior inhibition, and broad-spectrum neutralizing activity against MERS-CoV infection without negatively affecting expression level or stability.

Di-Nb and Tri-Nb had molecular weights of about 32 and 48 kDa that were double and triple the size of Mono-Nb (~16 kDa), respectively. As expected, engineering of the RBD-specific Mono-Nb had no impact on antibody expression. As with Mono-Nb, Di-Nb and Tri-Nb could be expressed at high levels in a yeast cell expression system and purified with high purity, retaining their conformation and antigenicity. In addition, the larger size of the oligomeric Nbs did not affect their stability. Like Mono-Nb, the oligomerized Di-Nb and Tri-Nb maintained stability under all extreme conditions tested, including acidic or alkaline pH, protease (pepsin), chaotropic denaturant (urea), and high temperature. By contrast, the protease, chemical, and thermal stability of MERS mAb significantly decreased after these treatments. Thus, unlike the mAb control, the engineered MERS-CoV RBD-specific Nbs developed in this study maintained the key characteristics of Nbs, including intrinsic stability, high expression, and intact conformation. Because these Nbs are stable under above extreme conditions, they can be transported and stored without the need for refrigeration or special care, a marked advantage relative to traditional antibodies. This will significantly simplify the transportation and therapeutic processes of antibodies, particularly in Middle Eastern countries that generally have inadequate transport services and high temperatures during summer.

The MERS-CoV RBD-specific dimeric and trimeric Nbs developed in this study also have several important features related to interference with three critical steps of MERS-CoV infection. First, they exhibited significantly greater ability than the Mono-Nb to bind MERS-CoV RBD proteins with or without RBD mutations from divergent MERS-CoV strains isolated from different years and hosts, facilitating the Nb–RBD interaction and increasing the time that the Nbs are bound to MERS-CoV. We have previously demonstrated that the Mono-Nb recognizes an epitope at residue around D539 of MERS-CoV RBD [41]. Second, relative to Mono-Nb, the engineered oligomeric Nbs strongly blocked RBD binding to MERS-CoV receptor DPP4, which is involved in a key step of viral entry and infection [14,56], thereby more effectively blocking entry of MERS-CoV into its target cells. Third, Di-Nb and Tri-Nb had significantly greater capacities to neutralize homologous MERS-CoV (EMC2012 strain), as well as potently cross-neutralize dozens of heterologous MERS-CoV strains harboring one or two mutations in their RBDs, all of which were isolated from different countries, hosts (human and camel), and time periods. Overall, the engineered oligomeric Nbs have great potential to neutralize new MERS-CoV strains with mutations in the RBD, and could therefore play a key role in preventing camel-to-human and human-to-human transmissions of MERS-CoV.

Despite the significant advantages of Nbs, it should be noted that the engineered dimeric (~32 kDa) and trimeric (~48 kDa) Nbs, as well as their monomeric counterpart (~16 kDa), are smaller than IgG antibodies (~150 kDa), and their molecular weights are far below the kidney filtration threshold (~60 kDa). Therefore, these small Nbs may be rapidly eliminated from the bloodstream by renal clearance, thus they might have shorter half-lives than mAbs [41,55]. One approach of increasing the in vivo half-life of Nbs is to fuse them with albumin-binding domain (ABD) or human Fc (hFc) [55,57,58]. We have also successfully fused monomeric Nb with a C-terminal hFc, increasing its half-life as well as its therapeutic and prophylactic efficacy against MERS-CoV infection [41]. In future studies, we plan to extend the half-lives of the constructed oligomeric Nbs by fusing them with ABD or hFc, and then compare their in vivo efficacy with that of Mono-Nb with or without hFc against MERS-CoV.

To summarize, in this study we developed oligomeric MERS-CoV RBD-specific Nbs and demonstrated that their in vitro activities were superior to those of their monomeric Nb counterparts, including antigen-binding affinity, inhibition of virus–receptor binding, and enhanced neutralizing and cross-neutralizing activity against variant strains of MERS-CoV infection, without reducing their stability under harsh conditions. These Nbs have the potential to be developed as therapeutics to prevent and treat MERS-CoV infection. Similar strategies could be applied to developing therapeutic agents against other emerging and re-emerging infectious viruses with pandemic potential.

## Figures and Tables

**Table 1:** Stability of MERS-CoV RBD-specific Nbs against extreme conditions.

Conditions	Treatment	ND_50_ (nM, Mean Value)				
		Mono-Nb	Di-Nb	Tri-Nb	MERS mAb	SARS mAb
pH	pH 5.0	1.94	0.21	0.03	0.53	ND
	pH 7.0	2.00	0.21	0.03	0.57	ND
	pH 8.0	1.93	0.21	0.03	0.53	ND
	No treatment	2.00	0.23	0.03	0.56	ND
Pepsin	0 µg/mL	2.23	0.29	0.04	0.56	ND
	25 µg/mL	2.24	0.27	0.04	1.19 **	ND
	625 µg/mL	2.20	0.26	0.04	1.86 **	ND
	No treatment	2.15	0.24	0.04	0.55	ND
Urea	0 mM	2.27	0.25	0.04	0.56	ND
	50 mM	2.01	0.29	0.04	0.85 **	ND
	400 mM	2.18	0.25	0.03	1.46 **	ND
	No treatment	2.19	0.25	0.04	0.54	ND
Temperature	4 °C	2.06	0.25	0.03	0.56	ND
	37 °C	1.80	0.22	0.04	1.32 **	ND
	60 °C	2.21	0.26	0.03	2.06 ***	ND
	No treatment	2.17	0.26	0.04	0.58	ND

Note: Nbs were tested for stability under extreme conditions, including pH, protease (pepsin), chaotropic agent (urea), and temperature. Nbs were treated at different pH values (pH 5.0, 7.0, and 8.0) for 24 h at room temperature, the indicated concentrations of pepsin for 1 h at 37 °C or urea for 24 h at 25 °C, and different temperatures (4 °C, 37 °C, and 60 °C) for 24 h, followed by measurement of their neutralizing activity against pseudotyped MERS-CoV (EMC2012 strain) infection. Neutralizing activity of Nbs is expressed as mean 50% neutralization dose (ND_50_) (*n* = 2). Experiments were repeated twice, yielding similar results. MERS mAb and SARS mAb were used as controls. Significant differences between treatment and no-treatment groups under each condition were compared by mean ND_50_ ± s.e.m. *, **, and *** indicate the level of significance of the differences between MERS mAb, with or without treatment under the indicated conditions. ND, not detectable.

**Table 2:** Source of divergent MERS-CoV strains and cross-neutralizing activity of MERS-CoV RBD-specific Nbs against these strains.

Accession No.	Isolate Year	Host	Country	S Protein RBD Mutation(s)	ND50 (nM, Mean Value)				
					Mono-Nb	Di-Nb	Tri-Nb	MERS mAb	SARS mAb
AFS88936	2012	Human	Saudi Arabia	—	2.14	0.24 **	0.03 **	0.57	ND
AGV08379	2012	Human	Saudi Arabia	D509G	3.39	0.19 ***	0.06 ***	122	ND
AGV08584	2012	Human	Saudi Arabia	V534A	6.64	0.38 *	0.08 *	2.02	ND
AFY13307	2012	Human	UK	L506F	27.1	3.29 ***	0.37 ***	67.5	ND
AHI48528	2013	Human	Saudi Arabia	A431P, A482V	1.10	0.10 ***	0.02 ***	0.52	ND
AHI48733	2013	Human	Saudi Arabia	A434V	6.67	0.11 *	0.05 *	2.11	ND
AHC74088	2013	Human	Qatar	S460F	2.57	0.26 **	0.04 **	0.50	ND
AKM76239	2013	Human	Oman	V514L	9.07	0.95 *	0.09 **	3.65	ND
AID55090	2014	Human	Saudi Arabia	T424I	0.81	0.07 **	0.01 **	0.80	ND
AID55087	2014	Human	Saudi Arabia	Q522H	1.49	0.09 ***	0.02 ***	0.28	ND
ALX27228	2014	Human	Jordan	E536K	14.7	2.96 **	0.61 **	5.54	ND
ALJ76277	2014	Human	Saudi Arabia	D537E	7.30	1.49 *	0.41 *	3.02	ND
ALJ54518	2015	Human	Saudi Arabia	L507P	13.1	2.76 ***	0.43 ***	186	ND
ALB08322	2015	Human	South Korea	D510G	2.28	0.23 *	0.04 **	8.92	ND
ALB08289	2015	Human	South Korea	I529T	3.77	0.24 ***	0.08 ***	865	ND
ATG84888	2016	Human	Saudi Arabia	S426R	12.6	1.60 **	0.34 ***	15.6	ND
AHY22545	2013	Camel	Saudi Arabia	K400N	1.77	0.20 ***	0.02 ***	1.12	ND
AHY22555	2013	Camel	Saudi Arabia	A520S	1.11	0.17 **	0.08 **	1.10	ND
ASU90076	2015	Camel	UAE	S460T	6.84	1.26 ***	0.26 ***	3.36	ND

Note: MERS-CoV strains were isolated in human and camel from 2012 to 2016 in different countries. EMC2012 (accession no. AFS88936) is the prototypic MERS-CoV strain. RBD mutations indicate mutant residues in the RBD of S protein of the indicated MERS-CoV isolates. A MERS-CoV neutralization assay was performed to test cross-neutralizing activity of MERS-CoV RBD-specific Nbs against pseudotyped MERS-CoV expressing the S protein of these strains. Neutralizing activity of Nbs is expressed as mean ND_50_ (*n* = 2). Experiments were repeated twice, yielding similar results. MERS mAb and SARS mAb were used as controls. Significant differences between Di-Nb and Mono-Nb, as well as between Tri-Nb and Mono-Nb, were compared by mean ND_50_ ± s.e.m. *, **, and *** indicate the level of significance of the differences between Di-Nb or Tri-Nb and Mono-Nb for the indicated MERS-CoV strains. ND, not detectable.
